# *Achromobacter* infections from a pediatric population in Qatar

**DOI:** 10.1128/spectrum.03674-25

**Published:** 2026-02-20

**Authors:** Mohammed Suleiman, Andrés Pérez-López

**Affiliations:** 1 Department of Pathology, Sidra Medicine 187187 , Doha, Qatar; 2 Department of Biomedical Sciences, Qatar University 61780, Doha, Qatar; 3 Department of Pathology and Laboratory Medicine, Weill Cornell Medicine-Qatar 36579 , Doha, Qatar; JMI Laboratories, North Liberty, Iowa, USA

**Keywords:** *Achromobacter*, pediatric, antimicrobial susceptibility

## LETTER

*Achromobacter* is an emerging opportunistic pathogen in the pediatric population. As a biofilm-forming pathogen, it is being identified with increasing frequency as a colonizer of the respiratory tract in pediatric patients with cystic fibrosis (CF) (1, 2). Furthermore, the isolation of *Achromobacter* in CF patients has been associated with frequent exacerbations, accelerated decline in lung function, and higher mortality (3, 4). It has also been associated with nosocomial outbreaks in pediatric onco-hematology units and neonatal intensive care units (5, 6). There are limited antimicrobial susceptibility data available for *Achromobacter* in children. We conducted a retrospective review of the antimicrobial susceptibility patterns of *Achromobacter* isolates recovered from patients under 18 years of age in the clinical microbiology laboratory of Sidra Medicine, Qatar’s national pediatric referral center, between September 2020 and 2025. All isolates were identified using matrix-assisted laser desorption/ionization time-of-flight mass spectrometry (MALDI-TOF MS) (Bruker, Bremen, Germany). Antimicrobial susceptibility testing (AST) was performed using the BD Phoenix M50 System (Becton Dickinson, New Jersey, USA), and minimum inhibitory concentrations (MICs) were interpreted according to the Clinical and Laboratory Standards Institute (CLSI) standards for “Other non-Enterobacterales” (7). A total of 122 isolates were analyzed during the 5-year study period. We were only able to identify isolates to the genus level as *Achromobacter* species due to the inability of our MALDI-TOF database to accurately differentiate individual species. Most strains (112; 91.8%) were isolated from respiratory specimens, including 34 isolates from CF patients (27.9%) (Table 1). In terms of AST, our isolates showed high susceptibility to imipenem (96.7%), meropenem (97%), piperacillin-tazobactam (100%), trimethoprim-sulfamethoxazole (93.4%), and ceftazidime (88.5%), which supports the use of these agents to treat *Achromobacte*r infections. There is a lack of pediatric studies, but several available studies in adults and CF patients are consistent with our findings and support the use of the same antibiotics as first-line treatment option (8, 9). In addition, our isolates showed low susceptibility to ciprofloxacin (11.5%), despite quinolones being seldom prescribed in children, and cefepime (11.5%), which supports the recommendation to avoid these agents in the empirical treatment of *Achromobacter* infections. It is also worth noting that isolates obtained from respiratory samples of our CF patients showed lower susceptibility to ceftazidime (70.6%) and levofloxacin (47%) but showed consistent results for the other antibiotics compared with non-CF specimens. In conclusion, *Achromobacter* is an emerging pathogen causing infections in selected pediatric populations like CF patients. Our findings support the use of trimethoprim-sulfamethoxazole, piperacillin-tazobactam, or carbapenems as initial therapy for *Achromobacter* infections in children. Further research evaluating the microbiological and clinical features of *Achromobacter* infections in the pediatric population is needed.

**TABLE 1:** Clinical characteristics and susceptibility data of *Achromobacter* isolates recovered from pediatric patients at Sidra Medicine, Qatar (2020–2025)^*b*^

Clinical characteristics for all isolates (*n* = 122)			
Age	0–18 years old, median = 3 years old		
Gender	68 males, 54 females		
Specimen type	112 respiratory (34 from CF patients), four urine, five ear, one CSF shunt		

Susceptibility data for all isolates (*n* = 122)			
Antibiotic	% Susceptible (MIC)	% Intermediate (MIC)	% Resistant (MIC)
Cefepime	11.5 (≤8)	18.1 (16)	70.4 (≥32)
Ceftazidime	88.5 (≤8)	10.7 (16)	0.8 (≥32)
Ciprofloxacin	11.5 (≤1)	19.7 (2)	68.8 (≥4)
Gentamicin	0 (≤4)	0 (8)	100 (≥16)
Imipenem	96.7 (≤4)	2.5 (8)	0.8 (≥16)
Levofloxacin	80.3 (≤2)	6.6 (4)	13.1 (≥8)
Meropenem	96.7 (≤4)	3.3 (8)	0 (≥16)
Norfloxacin	5 (≤4)	0 (8)	95 (≥16)
Piperacillin-tazobactam	100 (≤16/4)	0 (32/4–64/4)	0 (≥128/4)
Trimethoprim-sulfamethoxazole	93.4 (≤2/38)	0^*a*^	6.6 (≥4/76)

Susceptibility data for isolates from CF patients (*n* = 34)			
Antibiotic	% Susceptible (MIC)	% Intermediate (MIC)	% Resistant (MIC)
Cefepime	14.7 (≤8)	14.7 (16)	70.6 (≥32)
Ceftazidime	70.6 (≤8)	26.5 (16)	2.9 (≥32)
Ciprofloxacin	11.8 (≤1)	29.4 (2)	58.8 (≥4)
Gentamicin	0 (≤4)	0 (8)	100 (≥16)
Imipenem	100 (≤4)	0 (8)	0 (≥16)
Levofloxacin	47 (≤2)	23.5 (4)	29.5 (≥8)
Meropenem	97 (≤4)	3 (8)	0 (≥16)
Norfloxacin	0 (≤4)	0 (8)	100 (≥16)
Piperacillin-tazobactam	100 (≤16/4)	0 (32/4–64/4)	0 (≥128/4)
Trimethoprim-sulfamethoxazole	94.4 (≤2/38)	0^*a*^	5.6 (≥4/76)

^*a*^ No breakpoint available.

^*b*^ CF, cystic fibrosis; CSF, cerebrospinal fluid; MIC, minimum inhibitory concentration.
